# 

**Published:** 1816-05

**Authors:** 


### MONTHLY CATALOGUE OF MEDICAL BOOKS.

A Treatise on the Medicinal Leech, including its Medical and Natural History, with a Description of its Anatomical Structure: also, Remarks upon the Diseases, Preservation, and Management of Leeches. By J. R. Johnson, M.D. F.L.S. 8vo. boards.

A System of Mineralogy. By Robert Jameson, Professor of Natural History, &c. &c. &c. 3 vols. 8vo. boards.

A Compendium of Medical Practice, illustrated by interesting and instructive Cases, and by Practical, Pathological, and Physiological Observations. By James Bedingfield. Royal 8vo. boards.

---

### *NOTICES TO CORRESPONDENTS.*

*Communications have been received from* Dr. Walker, C. S. Gaye, Esq., Mr. Whitford, J. W., L. G., *&c. &c. which shall appear in our next.*

